# Establishment and characterization of pleomorphic adenoma cell systems: an in-vitro demonstration of carcinomas arising secondarily from adenomas in the salivary gland

**DOI:** 10.1186/1471-2407-9-247

**Published:** 2009-07-21

**Authors:** Satoshi Maruyama, Jun Cheng, Susumu Shingaki, Takashi Tamura, Shuichi Asakawa, Shinsei Minoshima, Yoshiko Shimizu, Nobuyoshi Shimizu, Takashi Saku

**Affiliations:** 1 Divisions of Oral Pathology, Department of Tissue Regeneration and Reconstruction, Niigata University, Graduate School of Medical and Dental Sciences, 2-5274 Gakkoucho-dori, Chuo-ku, Niigata 951-8514, Japan; 2 Oral Pathology Section, Department of Surgical Pathology, Niigata University Hospital, 1-754 Asahimachi-dori, Chuo-ku, Niigata 951-8520, Japan; 3 Divisions of Reconstructive Surgery for Oral and Maxillofacial Region, Department of Tissue Regeneration and Reconstruction, Niigata University, Graduate School of Medical and Dental Sciences, 2-5274 Gakkoucho-dori, Chuo-ku, Niigata 951-8514, Japan; 4 Department of Medical Genetics, School of Health Sciences, Kyorin University, 476 Miyashita-cho, Hachioji-shi, Tokyo 192-8508, Japan; 5 Department of Molecular Biology, Keio University School of Medicine, 35 Shinanomachi, Shinjuku-ku, Tokyo 160-8582, Japan; 6 Medical Photobiology Department, Photon Medical Research Center, Hamamatsu University School of Medicine, 1-20-1 Handayama, Hamamatsu 431-3192, Japan; 7 Advanced Research Center for Genome Super Power, Keio University, 2 Okubo, Tsukuba, Ibaraki 300-2611, Japan

## Abstract

**Background:**

Among the salivary gland carcinomas, carcinoma in pleomorphic adenoma has been regarded as a representative carcinoma type which arises secondarily in the background of a pre-existent benign pleomorphic adenoma. It is still poorly understood how and which benign pleomorphic adenoma cells transform into its malignant form, carcinoma ex pleomorphic adenoma.

**Methods:**

We have established five cell systems from a benign pleomorphic adenoma of the parotid gland of a 61-year-old woman. They were characterized by immunofluorescence, classical cytogenetics, *p53 *gene mutational analysis, fluorescence in-situ hybridization, and histopathological and immunohistochemical examinations of their xenografts, to demonstrate their potency of secondary transformation.

**Results:**

We established and characterized five cell systems (designated as SM-AP1 to SM-AP5) from a benign pleomorphic adenoma of the parotid gland. SM-AP1 to SM-AP3 showed polygonal cell shapes while SM-AP4 and SM-AP5 were spindle-shaped. SM-AP1-3 cells were immunopositive for keratin only, indicating their duct-epithelial or squamous cell differentiation, while SM-AP4/5 cells were positive for both keratin and S-100 protein, indicating their myoepithelial cell differentiation. Chromosome analyses showed numeral abnormalities such as 5n ploidies and various kinds of structural abnormalities, such as deletions, translocations, derivatives and isodicentric chromosomes. Among them, der(9)t(9;13)(p13.3;q12.3) was shared by all five of the cell systems. In addition, they all had a common deletion of the last base G of codon 249 (AGG to AG_) of the *p53 *gene, which resulted in generation of its nonsense gene product. Transplanted cells in nude mice formed subcutaneous tumors, which had histological features of squamous cell carcinoma with apparent keratinizing tendencies. In addition, they had ductal arrangements or plasmacytoid appearances of tumor cells and myxoid or hyaline stromata, indicating some characteristics of pleomorphic adenoma.

**Conclusion:**

This study demonstrates in vitro that certain cell types from pleomorphic adenoma are able to clone and survive over a long term and develop subcutaneous tumors in nude mice. The histological features of squamous cell carcinoma from the transplanted cell systems in nude mice might suggest a secondary onset of malignancy from a pre-existing benign adenoma.

## Background

Among the salivary gland carcinomas, carcinoma ex pleomorphic adenoma has been regarded as only one carcinoma type, which is considered to arise in the background of a pre-existent benign adenoma. The frequencies of the secondary onset of carcinoma have been recorded to be 6.2% to 8.8% among pleomorphic adenomas, although cellular mechanisms for how carcinoma cells develop in pleomorphic adenomas are poorly understood [1,2]. In our previous study, we proposed a concept of focal carcinomas in pleomorphic adenoma which is an advanced stage of accumulated atypical cells with P53 over-expressions as an initial stage or a latent form of apparent carcinomas secondarily arising in pleomorphic adenoma [3]. Although pathologists in their daily services of surgical pathology had recognized such singular atypical cells in pleomorphic adenomas, these atypical cells were not always regarded as evidence or the source for malignant transformation [4-6].

Pleomorphic adenomas have been often subjected to cytogenetic and molecular analyses. Among those studies, the PLAG1 (pleomorphic adenoma-related gene), which is located in 8q12, has been investigated most extensively. PLAG1 is consistently rearranged in pleomorphic adenoma by translocations t(3;8)(p21;q12) [7,8] and t(5;8)(p13;q12) [9]. These translocations have been regarded as one of the major responsible genetic events for the tumorigenesis of pleomorphic adenoma. As another important cancer-related gene, the *p53 *gene has been most extensively investigated in surgical samples of both benign pleomorphic adenoma, focal carcinoma in pleomorphic adenoma [3] and carcinoma ex pleomorphic adenoma [10-18], and mutations in the *p53 *gene have been considered to be responsible for the malignant transformation of pleomorphic adenoma [10-12].

There have been three trials in the literature to establish cell lines/systems from pleomorphic adenomas [19-21] and two from carcinoma ex pleomorphic adenomas [13,22], in addition to those from mere primary cultures [7-9,23-27]. Kondo *et al*. [19] established an epithelial cell line named Nagoya-78 from a benign pleomorphic adenoma of the lip and showed that the cells contained 62–65 chromosomes with plenty of abnormalities. They also transplanted the cells in hamsters, whose histological phenotypes were malignant myoepitheliomas, to generate tumors within a few weeks. Jaeger *et al*. [20] also established a cell line named AP2 from a benign pleomorphic adenoma of the parotid gland, which showed myoepithelial-like characteristics in a three dimensional culture. Another cell line from a palatal pleomorphic adenoma was HPA by Shirasuna *et al*. [21]. This cell line was revealed ultrastructurally to show a myoepithelial differentiation. These reports described malignant or transformed natures of the cells, while no definite histological characteristics of squamous cell carcinoma were demonstrated. Unfortunately, no further investigations for these cell lines after the initial reports have been conducted, nor has any attention been paid to the gene mutational events in the salivary gland adenoma-carcinoma sequence.

It is thus necessary to analyze the pathogenetic mode of the secondary onset of carcinomas in benign pleomorphic adenomas further *in vitro*, because most of the previous investigations have been limited to only surgical specimens and primary cultures. In the present study, our aim was to clone cell systems from a pleomorphic adenoma to characterize its transformed cells in various aspects. Since we were successful in establishing five cell systems after a long-period of primary culture from a benign pleomorphic adenoma, we analyzed these cells for cellular differentiation, chromosomal abnormality, *p53 *gene mutation, and histology of xenografted tumors in nude mice.

## Methods

### Tumor sample

A fresh tissue sample was obtained from a parotid gland tumor of a 61-year-old woman. The tumor, measuring 23 × 20 × 15 mm in size, was surgically removed with tumor-free margins. The surgical material was fixed in 10% formalin, cut into about 3–5 mm thick slices using whole-organ sectioning, and embedded in paraffin. Sections 5 μm thick were cut from the cut surfaces of the tumor specimens and stained with hematoxylin and eosin (HE). Macroscopically, the tumor was circumscribed with a fibrous capsule, and was grayish white in color, solid, firm and mucinous in its cut surface. There were no signs of local recurrence or metastasis during her three-year postoperative course. The experimental protocol for isolation and analyses of tumor cells was reviewed and approved by the Niigata University Graduate School of Medical and Dental Sciences Ethical Board. Prior to obtaining the tissue samples, our purpose and plan of the experiment were explained to the patient followed by her consent.

### Primary culture and cloning

A tissue slice obtained from the central and maximum cut surface of the surgical material was minced into small pieces. The tissue pieces were treated with 0.1% (v/w) collagenase (F. Hoffmann-La Roche Ltd., Basel, Switzerland) in Dulbecco's minimal essential medium (DMEM, Gibco, Invitrogen. Co., Carlsbad, CA, USA) containing 10% fetal calf serum (Gibco), 50 IU/ml penicillin and 50 μg/ml streptomycin (Gibco) in a 2 ml tube for 8 hrs at 37°C. Pass-through fractions from a nylon mesh filter were washed and plated in 2 ml of DMEM in 35 mm dishes and incubated at 37°C in humidified 5% carbon dioxide/95% air atmosphere. After two weeks, culture media were replaced with fresh ones and thereafter changed every 7 days. After one month, the cells, which had grown to confluence, were split into 25 cm^2 ^flasks. When aggregates of polygonal and bizarre epithelioid cells appeared in the background of spindle-shaped cells in the fourth passage, the cells were split and thereafter passaged every week. After four passages, the cells were served for cloning. The cells prepared as above were plated in 96-well microplates using a conventional method of dilution [28]. Wells with a single cell were observed every 24 hrs by microscope. The culture media were changed every 2 days. After the cells reached subconfluency in the wells, they were transferred into 24 well plates and maintained for up to 1 month until they reached subconfluent conditions, and then the cells were moved to 25 cm^2 ^flasks.

### Immunohistochemistry

All of the established cell systems were used for immunofluorescence studies. The cells were seeded at a cell concentration of 1.2 × 10^4 ^onto each well of chamber slides (Lab-Tek II, 4-well type, Nalge Nunc International, Naperville, IL, USA) and cultivated for 6 days. At day 6 after plating, the chamber slides were fixed and served for immunofluorescence experiments [29]. The primary antibodies consisted of rabbit polyclonal antibodies against human keratin (wide spectrum, Dako, Glostrup, Denmark, diluted at 1:25), and human S-100 protein (Dako, 1:100) and mouse monoclonal antibodies against human cytokeratin (CK) 14 (clone CKB1, IgM, 1: 100, Sigma Chemical Co., St Louis, MO, USA), calpoinin (CALP^1^, IgG_1_, 1:50, Dako), and P53 protein (IgG_2a_, specific to the transcription domain in the NH_2_-terminal region, clone Bp53-11, Progen Bioteknik GmbH, Heidelberg, Germany). The secondary antibodies were rhodamine-conjugated goat anti-rabbit or mouse IgGs or IgM (ICN Pharmaceuticals, Aurora, OH, USA, 1:50). For control studies, purified non-immune rabbit IgG or mouse IgG_2a _(Dako) were used instead of the specific primary antibodies. Xenografts of pleomorphic adenoma cell systems in nude mice were also examined immunohistochemically for perlecan, a basement membrane type heparan sulfate proteoglycan, and fibronectin by using rabbit polyclonal antibodies (diluted at 50 μg/ml, respectively) [29] and the rabbit Envision+/HRP system (Dako). For control studies on the antibodies, the primary antibodies were replaced with preimmune rabbit IgG.

### DNA Extraction

Cellular DNAs were extracted from cells in primary culture and from all of the five established cell systems by using a TRIzol system (Invitrogen). The cells were cultivated for 7 days up to their subconfluency in 25 cm^2 ^flasks, and 1 ml of TRIzol reagent was added to each flask. Total RNAs were then extracted from the cell lysate according to the manufacturer's instructions. After complete removal of the aqueous RNA phase, DNA was isolated from the interphase and phenol-phase. Following precipitation with 0.1 M sodium citrate in 10% ethanol, the precipitants were washed with 70% ethanol, and the pellets were air-dried briefly. The DNA samples dissolved in autoclaved water were stored at -20°C.

### Polymerase chain reaction (PCR)

Exons 5-7 of the *p53 *gene were PCR amplified for sequencing to examine mutational events in cells in primary culture and all of the cloned cell systems. For exon 5, a set of primers (forward, 5'-TTCAA CTCTG TCTCC TTCCT-3'; reverse, 5'-GACCT CTCTG CTGTC CCGAC-3') was used to generate a 323 bp fragment. For exon 6, a set of primers (forward, 5'-GCCTC TGATT CCTCA CTGAT-3'; reverse, 5'-AGAGA CCCTC CTCCC CAATT-3') was used for a 223 bp fragment. For exon 7, a set of primers (forward, 5'-CTTGC CACAG GTCTC CCCAA-3'; reverse, 5'-CGGTG AACGG TGGGA CGTGT-3') was used for a 453 bp fragment. After first denaturation at 94°C for 4 min, the experimental protocol for 35-cycle PCR was performed as follows: denaturation at 94°C for 1 min, annealing at 60°C for 1 min, extension at 72°C for 1 min, and termination with a final extension at 72°C for 7 min. Their sequence primer sets were from the nested primers used in the PCR experiments but with Texas red labeling at their 5'ends.

### Direct sequencing of PCR products

All the PCR products for exons 5, 6 and 7 of the *p53 *gene were subjected to direct sequencing by using Thermo Sequenase Primer Core Cycle Sequencing kits with 7-deaza-dGTP (GE Healthcare Ltd./Amersham, Buckinghamshire, UK). One reaction mixture contained 3 μl of pre-mixes (appropriate nucleotides/reaction buffer/Thermo Sequenase DNA polymerase), 1 μl of the template PCR products purified with GFX PCR DNA and Gel Band Purification kit (Amersham), and 2 μl (2 pM) of Texas red-labeled primers. After denaturation at 95°C for 2 min, the reaction mixture was placed on a thermal cycler for 25 cycles of denaturation at 95°C for 30 sec and annealing/extension at 60°C for 30 sec. The reaction products were dissolved in 3 μl loading buffer and concentrated with a vacuum desiccator. Then, 3 μl of samples for each lane were loaded on a gel [7% Long Ranger (Biowhittaker Molecular Applications, Rockland, ME, USA)/6.1 M urea/1.2 × TBE buffer (0.445 M Tris-HCl, 0.445 M boric acid, 0.01 M EDTA)]. The electrophoresis was performed in a fluorescent DNA sequencer (SQ-5500-S, Hitachi Ltd., Tokyo, Japan) and the sequencing data were analyzed by using the SQ-5500 analysis software ver. 3.03 (Hitachi).

### Chromosomal analysis

Primary culture cells in the fourth passages and all of the established cell systems in their subconfluent conditions were arrested for 3.5 hrs with 0.06 μg/ml colcemid in DMEM. They were washed two times with PBS and then removed with 0.05% trypsin at 37°C for 5 min. The cells were recovered from culture dishes with DMEM, suspended in 0.075 M KCl at 37°C for 30 min for hypotonic treatment, and then fixed in 1:3 acetic acid/methanol. The cell suspension was dropped onto glass slides to spread chromosomes from cells in the metaphase at room temperature under 50–55% ambient humidity. Slides were stained with 6% Giemsa solution in 0.067 M phosphate buffer (pH 6.8) for visualization of chromosomes. Metaphase spreads from 20 to 50 cells of the primary culture and each cell system were counted for chromosome numbers. Other slides were treated with 0.005% trypsin at 2°C for 8 min. They were washed and then stained with Giemsa solution for visualization of Giemsa (G)-banding. Ten to twenty cells each were analyzed for G-banded karyotypes in all of the cell systems. For control studies, peripheral blood lymphocytes from the patient were cultured in RPMI 1640 (Gibco) containing 10% fetal calf serum (Gibco), 50 IU/ml penicillin and 50 μg/ml streptomycin (Gibco) for 3 days, and then analyzed for chromosome numbers and G-banded karyotypes in the same way as described above.

### Fluorescence in-situ hybridization (FISH) analysis of human bacterial artificial chromosome (BAC) clones

The genomic clones encoding the break point regions around t(9;13)(p13;q12) were isolated from the BAC library [30] by the PCR screening method with primer sets flanking the regions of the two chromosomes by using the GenBank database http://www.ncbi.nlm.nih.gov/ as shown in Table 1. About 10 μl of the DIG-labeled BAC DNA was mixed in 2 μl of 10 mg/ml salmon sperm DNA (Roche), 2 μl of 10 mg/ml *E. coli *tRNA (Roche), 4 μl of 4 M CH_3_COONH_4_, and 50 μl of 100% ethanol. After storage for 1 hr at -80°C, the mixture was melted and centrifuged in 8 μl of the hybridization mixture consisting of 50% formamide, 10% dextran sulfate, 2 × SSC solution for each slide. After 2 μl of 10.5 mg/ml human placenta DNA (Sigma) was added, the mixtures were denatured for 10 min at 75°C, cooled down, and supplemented with 1 μl BSA. The mixture was pre-annealed for 30 min at 37°C before applying it to the slides. Slide-bound chromosomal DNA was denatured in a solution of 70% formamide in 2 × SSC for 6 min at 75°C on preheated slides for 2 hrs at 60°C and dehydrated in a 4°C ethanol series (70% and 100% ethanol for 5 min each). Ten microliters of the hybridization mixture were applied to each slide, and the slides were sealed with cover glasses. After overnight incubation at 37°C, the cover glasses were removed and the slides were washed once in 50% formamide/2 × SSC for 20 min at 37°C and twice in 2 × SSC for 10 min at 37°C, and then in TNT solution (0.1 M Tris-HCl/0.15 M NaCl/0.05% Tween 20) for 5 min at room temperature. The slides were further incubated with 100 μl of TNB solution (0.1 M Tris-HCl/0.15 M NaCl/0.5% blocking solution (Dainippon Pharmaceutical Co. Ltd., Osaka, Japan) to block non-specific reactions. The DIG-labeled probes were reacted with the anti-DIG mouse monoclonal antibody (Roche, 50 μl of 1 μg/ml IgG/4 × SSC) for 30 min at room temperature, rinsed three times in TNT solution for 5 min, and then treated with the secondary antibody (40 μl of 10 μg/ml Alexa flour 488-conjugated goat anti-mouse IgG (Molecular Probes, Inc., Eugene, OR, USA)). Finally, the FISH slides were counterstained with 50 μg/ml propidium iodide (PI, Vector Laboratories, Burlingame, CA, USA) for 30 min at room temperature. FISH images were obtained by an Olympus confocal laser scanning microscope Fluoroview 1000 (Olympus Co., Tokyo, Japan).

**Table 1:** BAC correlated with human data in the database and primer sequences for each BAC clone.

	Chromosomal location in human		Primer		
		
BAC clones		Accession number	Forward sequence	Reverse sequence	PCR-Product (bp)
1537E12	9p13.2	AL161445	TGCTTCTTGGAAATCAAGTCAAAGGGTTAT	CTGAATCTGGCACTTGGATTGTCTCCATTC	150
1405F1	9p13.1	AL138834	TCAATGTCCCTTTGACCATTTCCAAATTCA	TTGAAGTCCACAGCTGCTGTGCCTCTGAAA	202
1529G11	9p12	AL161448	CCCTAAGAGGACCTGCAATTCTTCCTTCAG	GTTTTGTGGACCTTGAAGTGCTATATGGAA	190
1011G7	13q12.11	AL161772	ACTGGAGAGAATTTACTTTTACTTATGGTA	TTGAGACTCACAGCCTGAAGGGATAAACAC	250
2037C1	13q12.12	AL356287	CATTTCTTTGCCCTATAGACCTGATTGAAA	CTGTGCTCCCTTCATATAGCTTGTCTTCCTA	173
1325C2	13q12.2	AL591024	GCAAAATCCAGGGGTAGAGCTGAGTTGTGA	CTGAGATGGATTCTGTATTTGCCTATTTAC	140
1213F4	13q13.1	AL136160	CATCAAATGCCGTTTGAAGATATGAAGATG	TTTGGGAGCATCTTGACAGAATCCCTTTGA	180

### Xenografts in nude mice

To determine *in vivo *tumorigenicities of the cloned cell systems, SM-AP cells were transplanted in nude mice. Cells in their 9th to 13th *in vitro *passages at a concentration of 2 × 10^6 ^in 0.3 ml culture media were injected into the lateral back wall of female BALB/c (nu/nu) mice at 4 weeks of age (2 mice each per cell system). The animals were housed in clean boxes in a sanitary and ventilated animal room and maintained under constant conditions (at 22°C and in a 12-hr light/dark cycle) with free access to sterilized solid food and autoclaved water. When tumors reached sizes of around 10–15 mm in diameter, they were surgically removed with the animals under ether anesthesia. The excised tumor tissues were fixed in 10% formalin for 24 hrs at 4°C and embedded in paraffin. Serial sections cut at 5 μm from the paraffin blocks were stained with hematoxylin and eosin, Masson's trichrome and immunoperoxidase for perlecan, fibronectin, keratin, S-100 and P53, and then examined histologically. The experimental research by using animals was reviewed and approved by the Niigata University Graduate School of Medical and Dental Sciences Ethical Board.

## Results

### Tissue sample histology

**Figure 1:**
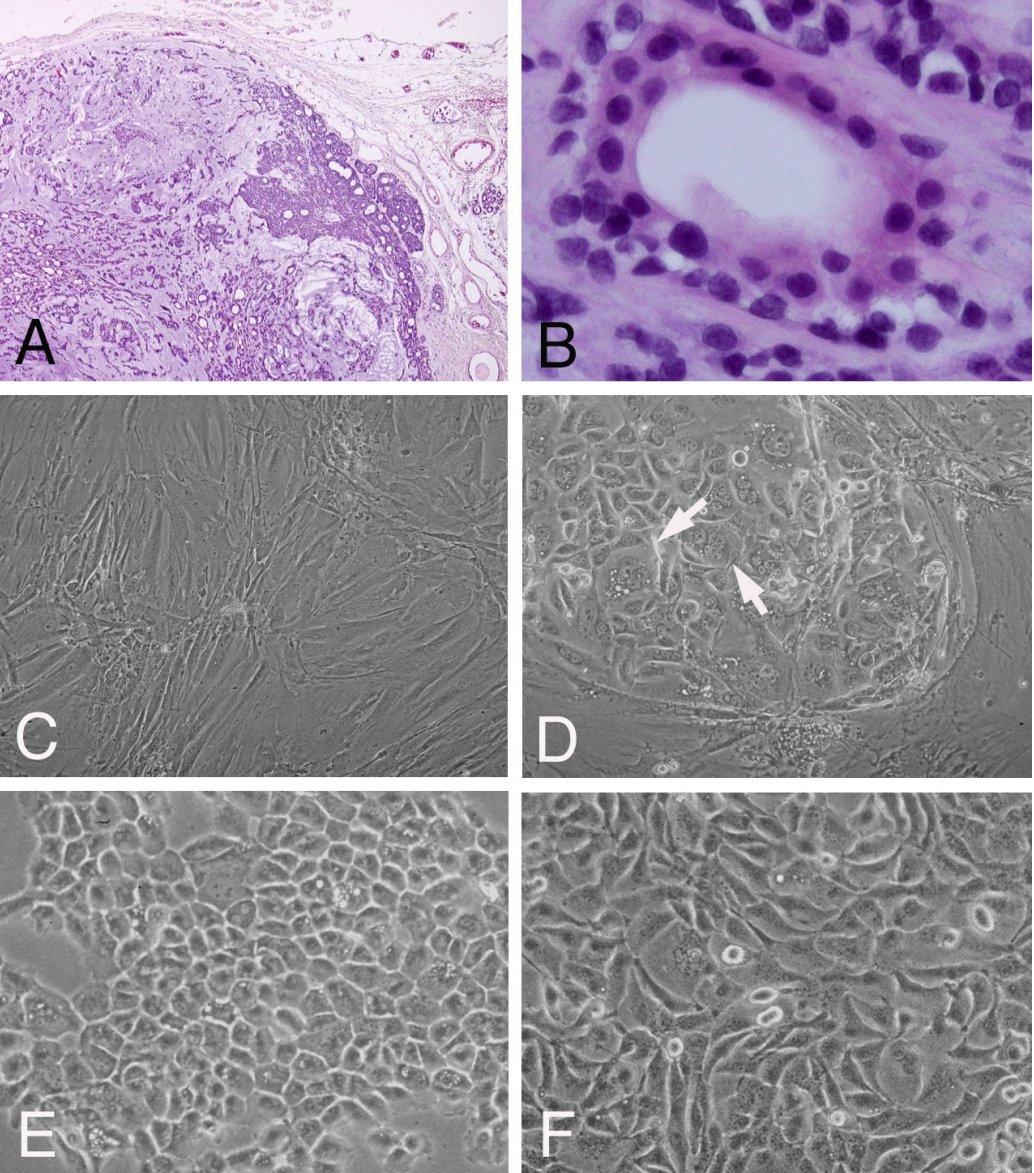
**Histopathology of pleomorphic adenoma from which cells were isolated (A, HE stain, × 40; B, HE stain, × 200) and phase-contrast microscopy of pleomorphic adenoma cells (C, primary culture, ×100; D, colony with bizarre cells, ×135; E, established cell system SM-AP3, × 150; F, SM-AP5, × 150)**. The surgical specimen showed typical features of benign pleomorphic adenoma (**A**), containing only a small number of atypical tumor cells (**B**). Cells in the primary culture were mainly spindle in shape mixed with fewer amounts of polygonal cells (**C**). In the fourth passage, aggregates of polygonal and bizarre epithelioid cells appeared in the background of spindle-shaped cells (**D**). From the following passage, five clones were successfully grown and isolated. They were classified into two groups according to their cell shapes. One was a polygonal shape represented by SM-AP3 (**E**), and the other was a short spindle shape one as shown by SM-AP5 (**F**).

The surgical specimen of the parotid gland tumor showed a typical histology of pleomorphic adenoma with a definite capsule (Figure 1A). The tumor contained both myxoid and hyaline areas with varied cellularity but poor vascularity. There were neither apparent foci of carcinoma cells nor pleomorphic adenoma cells with apparent atypical features (Figure 1B).

### Establishment of pleomorphic adenoma cell systems

For the first to third cell passages of the primary culture, it took about 2 months for each of the cells to form a confluent monolayer in a 25 cm^2 ^flask ready for splitting. During the period up to the third passage, cells were almost spindle-shaped in isolated colonies but did not show any fascicular modes of packing as seen in fibroblasts (Figure 1C). At the fourth passage, clusters of polygonal cells with ground glass-like cytoplasm started to appear among the spindle-shaped cells (Figure 1D), and the cells reached confluence in 7 days. After four additional passages, during which polygonal cells became enhanced in their atypical features in size and shape of their nuclei and such bizarre cells increased in number in most of the colonies, these cells were served for cloning. From serial dilutions in 96-well microplates, single cells were isolated in individual wells and grew to confluence within one month after plating. They were transferred into 24-well plates and then to a 25 cm^2 ^flask. These cloning procedures were repeated twice, and finally, 5 clones were successfully grown from the primary culture. They were designated as SM-AP1 to SM-AP5. After the cloning procedure, the five cloned cell systems were maintained by passages every 7 days. Their doubling times were approximately 31 hrs irrespective of the five cell systems. They were classified into two groups according to their cell shapes: one was polygonal with squamous epithelial characteristics, which was shared by SM-AP1 (Figure 1E), SM-AP2, and SM-AP3, and the other was short spindle shaped, which was characteristic of SM-AP4 and SM-AP5 (Figure 1F).

### Immunofluorescence of clones SM-AP cell systems

**Figure 2:**
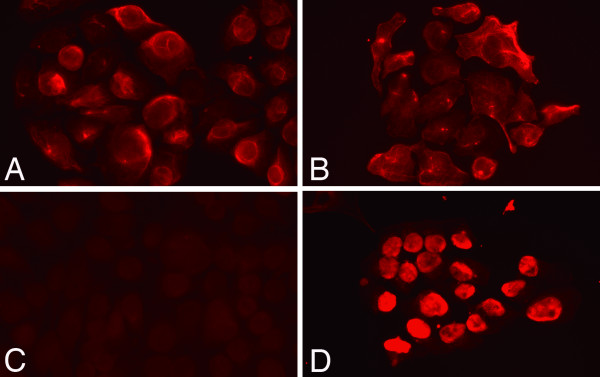
**Immunohistochemistry for keratin, duct epithelial marker (A-B) and S-100 protein, myoepithelial marker (C-D) in pleomorphic adenoma cell systems: SM-AP1 (A, C), SM-AP4 (B, D) at day 6 after plating, indirect immunofluorescence, × 200**. All of the cell systems, SM-AP1 to SM-AP5, were equally immunopositive for keratin (A-B). SM-AP1, SM-AP2, and SM-AP3 cells were not definitely positive for S-100 protein (C), while SM-AP4 and SM-AP5 cells were strongly positive for S-100 protein (D).

Clones SM-AP1 to SM-AP5 were stained positive for keratin, a duct-epithelial marker (Figures 2A–2D). SM-AP1, SM-AP2, and SM-AP3 cells were not positive for S-100 protein, a myoepithelial marker (Figures 2C), while SM-AP4 and SM-AP5 cells were strongly positive (Figures 2D) mainly in their nuclei. In addition to S-100 protein, SM-AP4 and SM-AP5 were positive for such myoepithelial markers as CK14 and calponin. All primary cultured cells and five cell systems showed immunopositivities for perlecan but none for P53 protein (not shown).

### Chromosome analysis

**Figure 3:**
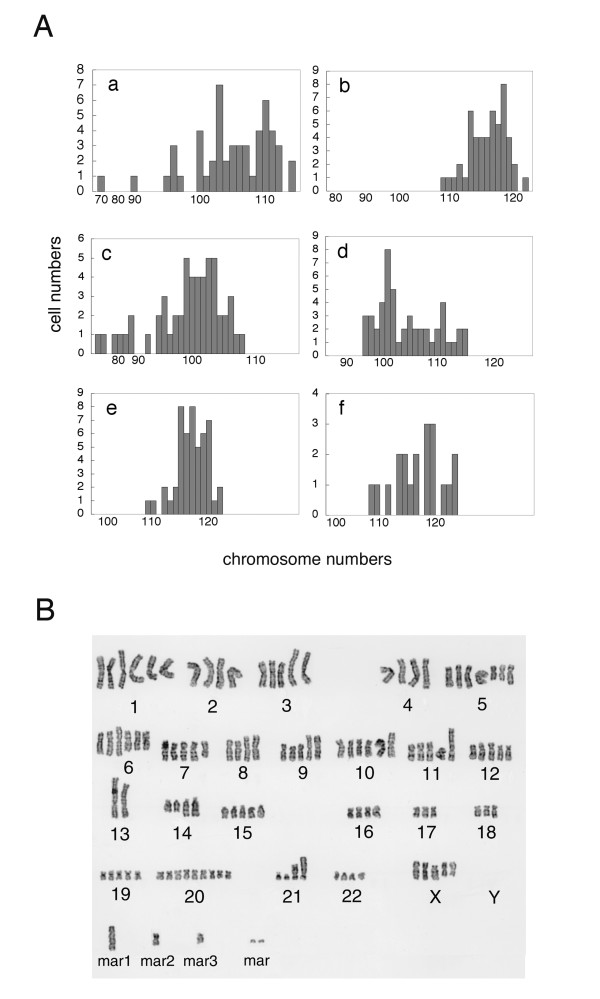
**Cytogenetic analysis of pleomorphic adenoma cell systems**. Panel **A**, chromosome numbers of pleomorphic adenoma cell systems: SM-AP1 (**a**), SM-AP2 (**b**), SM-AP3 (**c**), SM-AP4 (**d**), SM-AP5 (**e**), and those of cells in the primary culture (**f**) in histograms. Panel **B**, G-banded karyotyping of SM-AP5. Chromosome numbers of the five cell systems varied between 64 and 123, which were within tetraploid or pentaploid ranges, with a modal chromosome number of 108. Those of primary culture varied between 107 and 122 with a modal of 113.

Chromosome numbers of the five cell systems varied between 64 and 123, which were within tetraploid or pentaploid ranges, with a modal chromosome number of 108 (Figure 3A-a–e). Chromosome numbers of primary cultured cells varied between 107 and 122 with a modal chromosome number of 113 (Figure 3A-f). The G-banded karyotyping revealed a stemline karyotype of 102~114<5n>, XX in SM-AP1; 113~121<5n>, XX in SM-AP2; 98~108<5n>, XXX in SM-AP3; 97~115<5n>, XX, in SM-AP4; 114~121<5n>, XX in SM-AP5. Figure 3B represents the SM-AP5 stemline karyotype. All of the cell systems had numerous chromosomal abnormalities, such as deletion, translocation and marker chromosomes as shown in Table 2. Among them, additional materials of unknown origin such as add(X)(p11), der(3)add(3)(p11)der(3)(q2?), add(4)(q35) and translocations such as der(9)t(9;13)(p13;q12), add(12)(p11), and add(14)(q32) were shared by all of them. Since the der(9)t(9;13)(p13;q12) was the only abnormality in which the origin of translocated chromosomes was evident, this translocation was further analyzed for its break points by the FISH technique as described below. Chromosome 17, where the *p53 *gene is located at p13, decreased in number in all of the cell systems. In addition, del(17)(p11) was shared by four cell systems except for SM-AP4. SM-AP3 cells had add(17)(p13), which might have affected the *p53 *gene locus.

**Table 2:** Chromosome counts and stemline karyotyps of pleomorphic adenoma cell systems.

		stemline karyotypes	
		
cell systems	Cell numbers counted/karyotyped	Chromosome numbers <ploidy>	chromosomal abnomalities
SM-AP1	50/10	70–114 <5n>	XX, -X[6], -X[3], add(X)(p11)[10], add(X)[5], add(X)(q11)[7], +1[7], add(1)(p11)x2[10], add(1)(q11)[10],
			add(1)[9], -2[10], -2[10], +3[2], -3[5], der(3)add(3)(p11)del(3)(q2?)[10], der(3)add(3)del(3)[5],
			der(3)add(3)del(3)[2], -4[9], add(4)(p11)[2], add(4)(q35)[10], add(4)[8], +5[2], add(5)(q11)[10], add(5)[6],
			-6[9], -6[6], add(6)(q11)[8], -8[6], i(8)(q10)[10], i(8)[7], -9[3], der(9)t(9;13)(p13;q12)x2[10], -10[5],
			i(10)(q10)[9], -11[5], add(11)(p15)[9], +12[8], add(12)(p11)x2[10], add(12)[9], -13[10], -13[10], -13[4],
			add(13)(p11)[10], add(13)[4], -14[8], -14[3], add(14)(q32)[10], add(14)[9], +15[6], +16[2], -16[3],
			add(16)(q2?2)[10], add(16)[3], -17[4], del(17)(p11)[8], -18[9], add(18)(q11)[8], +19[8], +19[2], +20[8],
			+20[8], +21[10], add(21)(p11)[8], add(21)(p11)[5], der(21)t(13;21)(q11;p11)[7], -22[10], -22[9], -22[6],
			+mar1[9], +mar2[10], +mar2[5], +mar3[4], +mar5[2], +04mar.

SM-AP2	50/10	108–123 <5n>	XX, -X[6], add(X)(p11)[10], add(X)[6], add(X)(q11)[7], add(X)[2], +1[8], +1[2], add(1)(p11)x2[10],
			add(1)(p11)[2], add(1)(q11)[10], add(1)[7], -2[5], add(2)(p11)[7], idic(2)(q23)[7], -3[3],
			der(3)add(3)(p11)del(3)(q2?)[10], der(3)add(3)del(3)[6], -4[7], add(4)(q35)x2[10], +5[5], add(5)(q11)[9],
			add(5)[4], +6[3], add(6)(q11)[9], del(6)(q25)[3], -8[9], i(8)(q10)x2[10], +9[2], -9[3],
			der(9)t(9;13)(p13;q12)[10], der(9)t(9;13)[8], +10[7], +10[4], add(10)(p11)[4], i(10)(q10)[10], i(10)[7],
			add(11)(p11)[9], add(11)(p15)[10], +12[7], add(12)(p11)x2[10], add(12)[8], -13[10], -13[10], -13[10],
			-13[3], add(13)(p11)[10], add(13)[5], -14[7], add(14)(q32)x2[10], +15[6], -16[5], add(16)(q2?2)[8], -17[6],
			del(17)(p11)[4], -18[8], add(18)(q11)[8], add(18)(q21)[2], add(18)(q23)[5], +19[7], +20[9], +20[3], -21[8],
			add(21)(p11)[9], add(21)(p11)[10], add(21)(p11)[3], -22[10], -22[4],
			+mar1[8], +mar2[10], +mar2[2], +mar3 × 2[10], +mar4[2], +07mar.

SM-AP3	50/10	64–109 <5n>	XXX, -X[6], add(X)(p11) [10], add(X)(q11)[10], +1[8], add(1)(p11)x2[10], add(1)(q11)[10], add(1)[9],
			-2[10], der(3)add(3)(p11)del(3)(q2?)x2[10], -4[7], add(4)(q35)x2[10], -5[6], add(5)(q11)[10], add(5)[5],
			-6[9], -6[3], add(6)(q11)[7], -6[9], -6[3], add(6)(q11)[7], -7[9], -7[3], der(7;10)(q10;q10)[7], i(7)(q10)[2],
			-8[8], i(8)(q10)x2[10], -9[6], der(9)t(9;13)(p13;q12)x2[10], -10[7], -10[3], del(10)(p12)[2], i(10)(q10)[2],
			-11[6], add(11)(p11)[6], -12[5], add(12)(p11)[10], add(12)[3], -13[10], -13[10], -13[10], -13[9],
			add(13)(p11)[9], -14[9], -14[5], add(14)(q32)[10], add(14)[8], +15[4], -15[5], -16[9], -16[3],
			add(16)(q2?2)[8], -17[5], add(17)(p13)[8], del(17)(p11)[5], -18[9], -18[3], add(18)(q11)[10], add(18)[4],
			add(18)(q11)[2], add(18)(q21)[2], -19[6], +20[9], +20[8], +20[4], -21[9], -21[3], add(21)(p11)[10],
			add(21)(p11)[4], der(21)t(13;21)(q11;p11)[10], -22[10], -22[3],
			+mar1[8], +mar2[10], +mar2[7], +mar3 [8], +mar4[3], +04mar.

SM-AP4	50/10	97–115 <5n>	XX, -X[8], add(X)(p11)[10], add(X)(q11)[9], add(X)[2], +1[2], add(1)(p11)[9], add(1)[3],
			add(1)(q11)x2[10], -2[9], -3[7], der(3)add(3)(p11)del(3)(q2?)x2[10], der(3)add(3)del(3)[3], -4[10],
			add(4)(q35)[10], add(4)[9], +5[2], -5[5], add(5)(q11)[10], add(5)[3], -6[7], add(6)(p11)[5],
			add(6)(q11)[10], add(6)[5], +7[3], add(7)(q11)[4], -8[6], i(8)(q10)[10], i(8)[8], -9[5],
			der(9)t(9;13)(p13;q12)[10], der(9)t(9;13)[7], -10[6], i(10)(q10)[9], i(10)[2], -11[7], add(12)(p11)x2[10],
			-13[10], -13[10], -13[10],-13[4], add(13)(p11)[7], add(13)[2], add(13)(p11)[2], -14[9], add(14)(q32)[10],
			add(14)[9], der(14)t(1;14)(q11;p11), -16[7], -16[3], add(16)(q2?2)[7], add(16)[2], -17[10], -17[8], -18[10],
			-18[7], add(18)(q11)[9], add(18)[2], +19[5], +20[10], +20[9], +20[6], -21[10], -21[6], add(21)(p11)[9],
			add(21)(p11)[2], der(21)t(13;21)(q11;p11)[9], -22[10], -22[3],
			+mar1[7], +mar2[8], +mar3[7], +mar4[3], +mar5[2], +03mar.

SM-AP5	50/10	109–122 <5n>	XX, +add(X)(q11)[4], add(X)[9], add(X)(p11) [10], add(X)[9], +1[10], +1[3], add(1)(p11)[2],
			add(1)(p11)x2[10], add(1)(q11)[10], add(1)[8], i(1)(q10)[3], -2[10], -2[7], -3[4],
			der(3)add(3)(p11)del(3)(q2?)[10], der(3)add(3)del(3)[3], der(3)add(3)(p11)del(3)(q2?)[2], -4[10],
			add(4)(q35)x2[10], +5[9], add(5)(q11)x2[10], -6[8], add(6)(q11)[10], add(6)[2], -8[6], i(8)(q10)x2[10],
			i(8)[3], +9[3], add(9)(p11)[5], der(9)t(9;13)(p13;q12)[10], der(9)t(9;13)[9], +10[10], i(10)(q10)x2[10],
			-11[3], add(11)(p15)x2[10], add(11)[5], +12[5], add(12)(p11)x2[10], add(12)[5], -13[10], -13[10], -13[9],
			add(13)(p11)x2[10], -14[10], add(14)(p11)[3], add(14)(p11)[4], add(14)(q32)[10], add(14)[9], +15[5],
			+16[3], add(16)(q2?2)[10], add(16)[7], -17[8], -17[3], del(17)(p11)[3], -18[9],
			-18[3], add(18)(q11)[6], add(18)(q23)[6], del(18)(q21)[2], +19[10], +19[3], +20[10], +20[10], +20[2],
			-21[8], add(21)(p11)[10], add(21)(p11)[2], -22[9], -22[7], -22[3],
			+mar1[9], +mar2[10], +mar2[6], +mar3[8], +mar3[6], +mar5[2], +06mar.

Primary	20/20	107–122 <5n>	XX, -X, -X, -X, add(1)(p11), add(1)(q?12) x2, -2, -3, -3, add(4)(q31.3)x2, +5,
			der(5)add(5)(p15.1)add(5)(q22)x2, -6, add(6)(q21)x2, +add(7)(q11.2)x2, i(8)(q10)x2,
			-9, add(9)(p11), der(9)t(9;13)(p13;q12)x2, +10, add(10)(p11.1), i(10)(q10)x2, +11,
			add(11)(p11.2), add(11)(p15), +12, +12, add(12)(p11.2)x4, -13, -13, -13, -13, -14,
			add(14)(q?24)x2, +15, add(16)(q?12.1), -17, ? add(17)(p11.2)x2, -18, -18, +19, +20,
			-21, -21, -21, -22, +mar1 × 2, +mar2 × 2, +mar3 × 2, +mar4 × 2, +mar5 × 2 [cp20]

Primary cultured cells shared the same translocations as those of the established SM-AP cell systems, such as der(9)t(9;13)(p13;q12) and add(12)(p11) (Table 2). Peripheral blood lymphocytes from the patient had normal chromosome numbers and karyotype (not shown).

### Screening of chromosomal break points for translocation t(9;13)(p13;q12)

**Figure 4:**
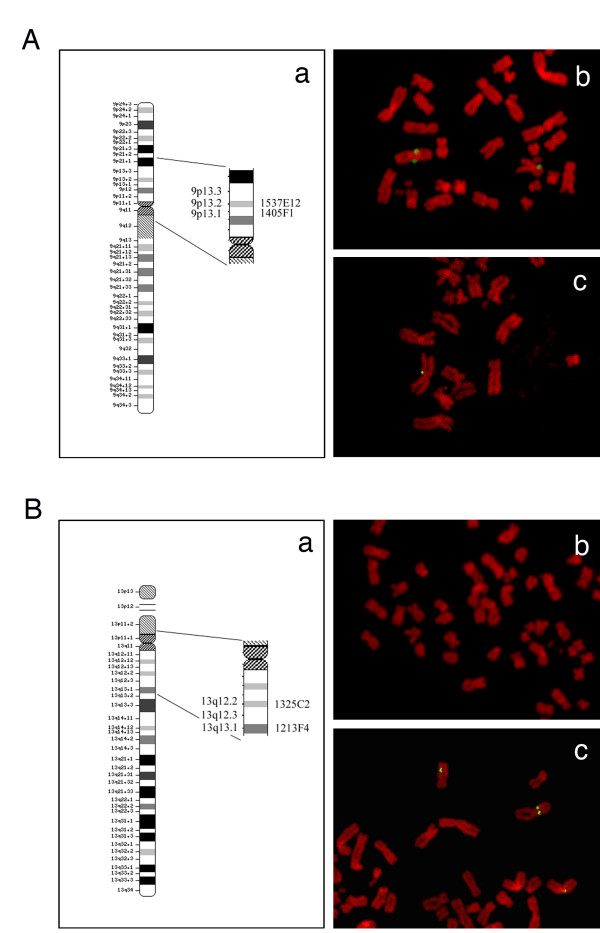
**Fluorescence in-situ hybridization (FISH) for screening of break points for the translocation, t(9;13)(p13;q12) in SM-AP5, by using BAC clones**. Panel **A**, mapping of BAC clones of chromosome 9 (**a**) and FISH for 9p13.2 by BAC 1537E12 (**b**) and for 9p13.1 by BAC 1405F1 (**c**). Panel **B**, mapping of BAC clones of chromosome 13 (**a**) and FISH for 13q12.2 by BAC 1325C2 (**b**) and for 13q13.1 by BAC 1213F4 (**c**). × 800. Signals of BAC clones, 1537E12 and1405F1 for chromosome 9p13.2 (A-b) and 9p13.1(A-c), 1325C2 for chromosome 13q12.2 (B-c) were detected as clear single and/or paired fluorescence dots on the translocation t(9;13)(p13;q12) chromosome, while no signal for BAC clone 1213F4 for chromosome 13q13.1 was seen (B-b).

To restrict the break points for the translocation t(9;13)(p13;q12), which was shared by all five cell systems, FISH analyses for chromosome 9p13 and 13q12 regions were performed. By using the primer sets listed in Table 1, three clones for chromosome 9 (p13) and four clones for chromosome 13 (q12) were isolated by PCR to map their chromosomal locations. Clear single or paired FISH signals were demonstrated by the BAC clones, 1537E12 (Figure 4A-b) and 1405F1 (Figure 4A-c), which are located in chromosome 9p13.2 and 9p13.1, respectively (Figure 4A-a). BAC clones by 1325C2 in chromosome 13q12.2 (Figure 4B-a) were localized on chromosomes with translocation t(9;13)(p13;q12) (Figure 4B-c). FISH signals for 1213F4, which is located in chromosome 13q13.1, were not found on any chromosomes (Figure 4B-b). Thus, break points for the translocation, t(9;13)(p13;q12), were thought to be located in restricted regions of 9p13.3 and 13q12.3.

### P53 gene analysis

**Figure 5:**
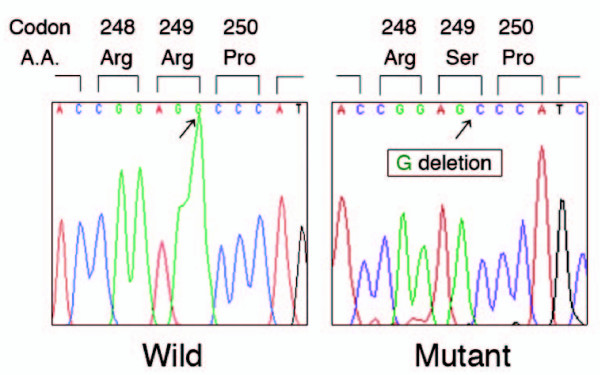
**Mutational analysis of the *p53 *gene in genomic DNA from pleomorphic adenoma cells in the primary culture as well as from the five SM-AP cell systems**. PCR-amplicons for exons 5, 6 and 7 of the *p53 *gene were directly sequenced, and the deletion of the last base G of codon 249 (AGG to AG-) in exon 7 was shared by all of the cell systems and the primary culture.

PCR amplicons for exons 5, 6 and 7 of the *p53 *genes from the primary cultured cells as well as from all of the SM-AP cell systems were sequenced, and deletion of the last base G of codon #249 (AGG to AG_) of exon 7 was found in all of them including the primary culture (Figure 5). Owing to this point mutation, a nonsense polypeptide should have been generated on and after codon #249, resulting in non-functional *p53 *gene products. No other mutations were found in exons 5 and 6.

### Xenografts of SM-AP cells in nude mice

**Figure 6:**
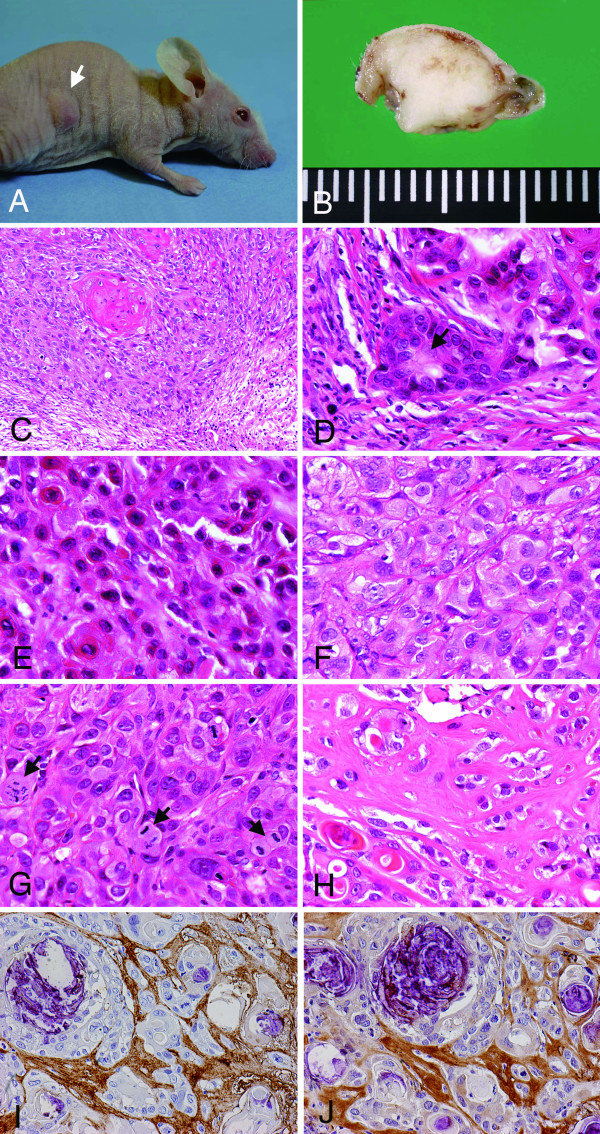
**Transplanted tumors of SM-AP cell systems in nude mice**. Macroscopic view of a tumor mass by SM-AP5 in lateral back in a nude mouse (**A**); cut surface view of a subcutaneous tumor by SM-AP1 (**B**); histopathology of transplanted tumors by SM-AP4 (**C**), SM-AP1 (**D, E**), SM-AP5 (**F**, **G**), and SM-AP3 (**H**). HE stain, C, × 100; D-G, × 320; H, × 240; immunoperoxidase stains of SM-AP3 transplants for perlecan (**I**) and fibronectin (**J**), × 200, hematoxylin counterstain. SM-AP cells formed subcutaneous tumors measuring about 10 mm in diameter in nude mice within one to four months (A). The tumors were rather limited to the dermis expanding into the superficial part of the muscle layer but had no capsular structure (B). Histopathologically, the tumors were basically squamous cell carcinomas with definite tendencies towards keratinization with invasive natures, although there was no basal cell alignment along the periphery of the tumor cell nests (C). Around the tumor cell nests, myxoid stroma was induced. SM-AP1 to SM-AP3 cells formed mimics of ductal structures (D), and at the same time, SM-AP1 and SM-AP2 showed plasmacytoid appearances (E). SM-AP4 and SM-AP5 cells formed less differentiated carcinomas composed of tumor cells with ground-glass-like cytoplasm (F). Irrespective of tumors, mitotic figures were frequently observed (G), and the stromata were wide, hyaline, and poor in vascularity and lymphocytic infiltration (H). The hyaline stroma was immunopositive for perlecan (I) and fibronectin (J).

Five cell systems, SM-AP1 to SM-AP5, successfully formed subcutaneous tumors in nude mice after 5 to 19 weeks of injections (Figure 6A, Table 3). The tumors which had grown to 10 to 15 mm in diameter were harvested for histopathological examinations (Figure 6B). Histologically, transplanted tumors showed rather expansive growths mostly limited to the range of the dermis to the subcutis or to only the surface part of the muscle layer, although they had no definite fibrous capsules. Tumor cell nests varied in size, and some of them contained keratinization centers, irrespective of transplanted tumors. Hence, squamous cell carcinomas were basically diagnosed for all of the tumors formed by the five cell systems. However, their keratinization seemed to occur suddenly without gradual differentiation tendencies in the tumor cell nests, which had no basal cell alignment at the periphery of the tumor cell nests (Figure 6C), which were usually not seen in squamous cell carcinomas of the oral mucosal origin. In the transplanted tumors of SM-AP1 to SM-AP3, which had been regarded as duct-epithelial by immunofluorescence *in vitro*, there were tendencies to form mimics of ductal structures (Figure 6D, arrow). Those of SM-AP1 and SM-AP2 contained tumor cells with plasmacytoid appearances (Figure 6E). In those of SM-AP4 and SM-AP5, which had rather myoepithelial natures *in vitro*, tumor cell nests were sheet-like with some tendencies toward keratinization, and they showed ground glass-like cytoplasm (Figure 6F). Mitotic figures were observed in each tumor but more frequently in SM-AP4 and SM-AP5 (Figure 6G, arrows), suggestive of their malignant natures. Hyaline stromal spaces without definite vascular channels and lymphocytic infiltration were formed between tumor cell nests in every tumor, which were also unusual in oral squamous cell carcinomas (Figure 6H). Immunohistochemically, the hyaline stroma was strongly positive for perlecan (Figure 6I) as well as for fibronectin (Figure 6J). Although there was no area with a typical histology of benign pleomorphic adenoma in the transplanted tumors, the presence of ductal mimics, plasmacytoid cells, ground-glass appearance of tumor cell cytoplasm and predominant hyaline stroma mentioned above might indicate their pleomorphic adenoma origin. However, there were no foci of salivary duct carcinoma, which is common in carcinoma ex pleomorphic adenoma. Immunohistochemically, the transplanted tumors of SM-AP1 to SM-AP5 were stained positive for keratin. SM-AP1, SM-AP2, and SM-AP3 cells were not positive for S-100 protein, while SM-AP4 and SM-AP5 cells were strongly positive for other myoepithelial markers, such as CK14 and calponin, in addition to S-100 protein. All five transplanted cell systems showed no P53 protein immunopositivities (not shown).

**Table 3:** Tumorgenicity of pleomorphic adenoma cell systems.

cell systems	Number of mice with tumors (n = 2)	Mean time of tumor appearance (weeks)
SM-AP1	2	7.5
SM-AP2	1	9
SM-AP3	1	19
SM-AP4	1	5
SM-AP5	2	9

## Discussion

In the present study, we were successful in establishing the five cell systems, SM-AP1 to SM-AP5, from a benign parotid gland pleomorphic adenoma. We previously suggested a potential of atypical tumor cells scattered in benign pleomorphic adenomas to develop into focal carcinomas and then into tangible forms of carcinoma ex pleomorphic adenoma [3], although it had been questionable whether the presence of these atypical cells within pleomorphic adenoma could be recognized as sources for the secondary onset of malignancy [4-6].

There have been three trials in the literature to establish cell lines/systems from pleomorphic adenomas in which only their malignant or transformed natures of the cells were reported with no definite histological characterization as squamous cell carcinoma [19-21], and two were from carcinoma ex pleomorphic adenomas [13,22], in addition to those from mere primary cultures [7-9,23-27]. In terms of cell shapes in culture, the cell system by Bullerdiek *et al*. was spindle [22], and CaPA-4 cells by Fujioka *et al*. [13] were squamous epithelial. In our immunohistochemical study, polygonal-shaped SM-AP1 to SM-AP3 were shown to be duct-epithelial, while spindle-shaped SM-AP4 and SM-AP5 were myoepithelial. In addition, their xenografts presented some ductal (SM-AP1-SM-AP3) or myoepithelial (SM-AP1 and SM-AP2 by plasmacytoid appearances) differentiation.

Interestingly, the two cell lines from carcinoma ex pleomorphic adenoma [13,22] were demonstrated to have characteristics of squamous cell carcinoma when they were transplanted into nude mice, as was also observed in the present study. Although our cell systems lost benign features of pleomorphic and had definite tendencies towards keratinization in xenografts, their histology was not always typical as seen in oral mucosal squamous cell carcinomas ones in terms of their cytoplasm and stroma. SM-AP cells had their characteristic cytoplasm with ground-glass appearances, and their hyaline or myxoid stromata were rich in perlecan and fibronectin and poorly vascularized. These features indicated that they were of pleomorphic adenoma origin. It took longer periods for them to form transplanted tumors, and they had no ulceration or metastasis, indicating that they were not so aggressive.

The five pleomorphic adenoma cell systems cloned in the present study showed aneuploid karyotypes and various kinds of chromosomal abnormalities, of which the translocation, der(9)t(9;13)(p13;q12), was stably shared by all of the clones. As a chromosome 9-related alteration, a reciprocal translocation t(9:12)(p13-21;q13-15) was found in benign pleomorphic adenomas by Mark *et al*. [24]. In the present study, we were able to restrict the break points of t(9;13)(p13;q12) within 9p13.3 and 13q12.3 in these cell systems. The result indicates that genes located in the distal region of 9p13.3 and the proximal region of 13q12.3 ares missing. The distal region of 9p13.3 is known to contain interferon α cluster (IFNA), a tumor suppressor gene (9p22) [31], methylthioadenosine phosphorylase (MTAP) (9p21) [32], and p16 (9p21) [33], and the break point at 13q12.3 contains IFN-inducible gene, namely the IFI-56K [34]. A role of p16 gene in the secondary onset of malignancy in pleomorphic adenomas has been hypothesized by Suzuki & Fujioka [33]. Since 9p13 rearrangements seem to be generated at the stage of pleomorphic adenoma, pleomorphic adenoma could be regarded as substantially malignant in nature even if it has a benign histological feature. The deletions of 9p22 containing IFNA and 9p21 containing MTAP and p16 genes and 9p allelic losses were also observed in oral squamous cell carcinoma [33,35-37].

None of the five pleomorphic adenoma cell systems in the present study showed any of these translocations involving 8q12, where PLAG1, one of the most extensively investigated genes in pleomorphic adenomas, is located. Astrom *et al*. [8] claimed that the 8q12 abnormalities are not a requirement for the enhanced expression of PLGA1 in pleomorphic adenoma by using primary cultures of both pleomorphic adenoma and carcinoma ex pleomorphic adenoma with or without 8q12 abnormalities. Abnormalities such as t(6,8)(p21-23;q12) [23], t(9;12)(p13-21;q13-15) [24], del(5)(q22-23;q32-33), t(10;12)(p15;q14-15) [38], and t(12;?)(q13-15;?) [22] found in our cell systems constitute a new finding in pleomorphic adenoma.

Loss of heterozygosity (LOH) has been demonstrated, by using surgical materials, at chromosome arms 8q (52%), 12q (28%), and 17p (14%) in benign pleomorphic adenomas and in adenoma components of carcinoma ex pleomorphic adenoma, whereas the ratios of LOH in 8q, 12q and 17p loci were enhanced up to 69%, 50%, and 69%, respectively, in carcinoma ex pleomorphic adenoma [39]. In our present result, pleomorphic adenoma cell systems tended to decrease numbers of chromosome 17 and abnormalities of add(17)(p11) and add(17)(p13). These chromosomal abnormalities may affect transcription of the *p53 *gene, which is located in 17p13.

The present result demonstrating that *p53 *gene products were not detectable immunohistochemically in the five cell systems both in culture and xenografts are consistent with the data from CaPA-4 cells [13]. In the previous reports, immunohistochemical expressions of P53 protein were not always obtained in tumor cells or in the cases examined (3% to 41% for pleomorphic adenoma cases and 41% to 75% for carcinoma ex pleomorphic adenoma cases) [10,11,15,16]. There may be two interpretations for the non-immunopositivity for *p53 *gene products in our cell systems: one is that the protein expression levels were lower than the sensitivity of the method, and another is that mutated gene products could not be recognized by the antibody, Bp53-11. The latter seems to be more likely in our case because the point mutation in codon #249 in exon 7, which was shared by all of the cell systems, should have generated a nonsense gene product, which could not be recognized by the antibody whose antigenic site is the transcriptional transactivation domain within the NH_2_-terminal region [40]. Since we confirmed that this point mutation at codon #249 even in cells in the primary culture might have existed before cloning transformed SM-AP cells, this suggests that this mutation plays a role in the tumorigenesis of benign pleomorphic adenoma as well as in its malignant transformation. Interestingly, the G to A transitional point mutation at the next codon #248 found in CaPA-4 cells was also found in the surgical tissue samples from the adenoma portion as well as from the carcinoma portion of their original carcinoma ex pleomorphic adenoma [13]. Thus, the mutations of the *p53 *gene should be considered to be an early event in the malignant transformation.

## Conclusion

The present data suggest that pleomorphic adenoma contains cells with genetic alterations even when its histology is benign and that carcinoma cells may develop from some of the population of benign forms. Whether the atypical cells within benign pleomorphic adenoma [3] can be the direct source for malignant transformation is hard to say. Since all of the xenografted tumors in nude mice were histologically squamous cell carcinomas, the present establishment of pleomorphic adenoma-derived squamous cell carcinoma cells can be regarded as an *in-vitro *demonstration of secondary development of malignancy from a benign adenoma. This process may correspond to the clinical form of carcinoma ex pleomorphic adenoma. However, it may be possible at least to speculate that under the circumstances, in which atypical cells are generated, pleomorphic adenoma cells attain some background for the secondary onset of malignancy, which may be generated from the combination of the *p53 *gene mutation and other chimera genes resulting from the specific translocations involving der(9)t(9; 13)(p13.3; q12.3) and other chromosome abnormalities.

## Competing interests

The authors declare that they have no competing interests.

## Authors' contributions

SM carried out cell culture, establishment of cell systems and molecular genetic studies and participated in preparation of the manuscript. JC carried out immunohistochemistry. SS carried out tumor tissue sampling. TT carried out chromosomal analysis. SA participated in sequencing. SM, YS, and NS participated in the design of fluorescence in-situ hybridization (FISH) analysis of human BAC clones. TS conceived the study and participated in its design and coordination and in preparation of manuscript. All authors have read and approved the final manuscript.

## Pre-publication history

The pre-publication history for this paper can be accessed here:

http://www.biomedcentral.com/1471-2407/9/247/prepub
